# Air Pollution, Blood Lipids, And Cognitive Performance From The Multi-Ethnic Study Of Atherosclerosis (MESA)

**DOI:** 10.1093/geroni/igaf122.2286

**Published:** 2025-12-31

**Authors:** Xi Pan, Robyn McClelland, Lilah Besser, Jana Hirsch, Joel Kaufman, Jing Cao, Timothy Hughes

**Affiliations:** Texas State University, San Marcos, Texas, United States; University of Washington, Seattle, Washington, United States; University of Miami, Boca Raton, Florida, United States; Drexel University, Philadelphia, Pennsylvania, United States; University of Washington, Seattle, Washington, United States; UT Southwestern Medical Center, Dallas, Texas, United States; Wake Forest University School of Medicine, Winston-Salem, North Carolina, United States

## Abstract

The current study aimed to identify the role of blood lipids in the air pollutant-cognition relationship. The study sample consisted of 1,518 adults aged 45+ in the Multi-Ethnic Study of Atherosclerosis (MESA). Outdoor residential concentrations of PM2.5, NO2, and NOx between Exams 1 (2000-2002) and 5 (2010-2012) were assessed using validated spatiotemporal models. Outcomes were three cognitive test scores of Cognitive Abilities Screening Instrument (CASI), the Digit Symbol Coding (DSC), and the Digit Span (DS) tests at Exam 6 (2016-2018). Total cholesterol (TC) and low-density lipoprotein cholesterol (LDL-C) concentrations in blood at Exam 5 were evaluated as mediators or modifiers using linear regression models. The inverse odds ratio weighting approach was used for mediation analyses. Models assessed gender and race/ethnicity related heterogeneity in air pollutant-cognition and blood lipid-cognition associations. The direct effect of NO2 and NOx on DSC scores was significant (ß=-2.96 for NO2; ß=-3.20 for NOx, per interquartile range increase), but there was no evidence of mediation by cholesterol levels. Interaction terms of air pollutants and LDL-C were significantly associated with CASI scores (ß = 0.02, p = 0.01 for PM2.5; ß = 0.03, p < 0.001 for NO2; ß = 0.02, p < 0.01 for NOx). Associations of DSC scores with NO2 and NOx were only significant among Black participants (ß = -7.11 for NO2; ß = -6.18 for NOx). Findings of the study suggest that air pollution monitoring and control may be effective for protecting brain health and delaying the onset of cognitive impairment and dementia in late adulthood.

